# Impact of different capping materials extracts on proliferation and osteogenic differentiation of cultured human dental pulp stem cells

**DOI:** 10.1038/s41598-025-93759-y

**Published:** 2025-04-01

**Authors:** Nihal A. Sultan, Hamdi H. Hamama, Mohammed E. Grawish, Radwa I. EL-Toukhy, Salah Hasab Mahmoud

**Affiliations:** 1 Conservative Dentistry Department, Faculty of Dentistry, Mansoura University, Mansoura, Egypt; 2 Oral Biology Department, Faculty of Dentistry, Mansoura University, Mansoura, Egypt

**Keywords:** Dental materials, Dental treatments, Endodontics, Restorative dentistry

## Abstract

This study aimed to evaluate the effects of four bioactive capping material extracts on the proliferation and osteogenic differentiation of human dental pulp stem cells. Four capping material extracts were evaluated in this study [Harvard MTA (calcium silicate), Retro MTA (calcium zirconia complex material), Activa Bioactive Base/Liner (bioactive glass-based material) and an experimental MCP-based pulp capping material). The materials prepared according to their manufacturers’ instructions in the form of discs. Each material disc was placed into one insert of a 6-well plate and covered with Dulbecco’s Modified Eagle Medium to produce extracts at 1:1 ratio. Human dental pulp stem cells represent the negative control group, cells cultured in osteogenic media represent the positive control group, while cells cultured on the tested extracts represent the test groups. Each specimen was assessed in triplicate by three independent assays. The proliferation of stem cells was evaluated via MTT assay and cell viability was determined by measuring optical density. Osteogenic differentiation was assessed via the alizarin red stain test by measuring the H-score and calcium concentration. The proliferation and osteogenic differentiation data were analyzed using one-way ANOVA followed by Tukey’s post hoc multiple comparison test (*p* ≤ 0.05). Regarding MTT assay results, osteogenic media was significantly greater than calcium zirconia complex and MCP-based material. In comparison with negative control and calcium zirconia complexes, calcium silicate significantly increased the optic density. Alizarin red staining revealed significantly low H-scores and calcium concentrations in the four tested capping materials in comparison with control group. The calcium concentration of calcium silicate material was significantly greater than the remaining tested materials.Calcium silicate-based materials seem to have the most reliable performance concerning the proliferation and osteogenic differentiation of human dental pulp stem cells. Newly introduced resin-based materials have shown acceptable results but need further investigation. The present study had a few limitations; mainly the need to perform more laboratory evaluations and in vivo studies.

## Introduction

The ultimate goal of restorative intervention is to maintain pulp vitality. Vital pulp therapy is referred to as direct pulp capping (DPC) and is considered as one of the most conservative therapeutic treatment modalities for accidentally traumatized dental pulp. This technique was introduced to induce favorable wound healing by stimulating reparative dentin formation at the exposure site^1^. Several materials have been utilized to cover traumatized pulp tissues and most of their manufacturers claim to categorize them as bioactive materials. These materials induce the formation of natural tooth calcific barriers to protect the exposed pulp against further irritation from the surrounding environment. Furthermore, they are assumed to stimulate the healing process through several anti-inflammatory functions^2^.

Pulp exposure triggers a pulpal response. In the first few minutes, large myelinated nerve fibers and small nonmyelinated fibers are destroyed, causing the release of neuropeptides. These neuropeptides are responsible for sudden increases in vascular permeability and vasodilatation^3^. Erythrocytes, inflammatory cells and undifferentiated mesenchymal dental pulp stem cells are found next to the exposure site^4^.

Human dental pulp stem cells (hDPSCs) are morphologically similar to fibroblasts with superior proliferative ability than traditional mesenchymal stem cells driven from the bone marrow. They can be stimulated to differentiate into precursors of various cell types. Consequently, hDPSCs are considered as a viable cell sources for different regenerative applications^5^.

There is a wide variety of DPC materials with different compositions and handling methods. Fast-setting synthetic calcium silicate cement has a working time of 2 min and a setting time of 40 min. The next restorative step can be performed after 5 min^6,7^. Hydraulic calcium zirconia complex material has a fast-setting reaction that has an initial setting time of 150 s. This material has biological features resembling MTA without its drawbacks (long sitting time and discoloration)^8,9^. Bioactive glass-based material was developed to provide physical properties and esthetics of resin composite materials accompanied with active ion release of glass ionemer (GI) materials. This material has 3 setting mechanisms. It is set with a light curing mechanism, acid-base reaction of GI and composite self-cure setting reaction^10^.

Calcium phosphate-based materials are classified as bioactive materials that have a great potential for stimulating bone growth and osteointegration in both dental and orthopedic implants^11^. They also have the advantage of being highly biocompatible. So, they have great potential to be successful DPC materials and induce the repair of exposed pulp^12^. According to the manufacturer, experimental PulpCap with 2% monocalcium phosphate (MCP) is a light-cure urethane-based resin with a modified calcium phosphate component that is active in the oral environment.

Different studies compared the biological properties of calcium silicate-based materials with those of light-cured calcium silicate material and calcium hydroxide (CH); it was concluded that calcium silicate material had a more favorable outcome^2,13,14^. Mineral trioxide aggregate (MTA) effect on cell viability and cell apoptosis of stem cells was examined, and it was suggested that MTA might have a cytotoxic effect when placed directly on the pulp^15^. In addition, laboratory study^7^ investigated the proliferation, adhesion and osteogenic differentiation of hDPSCs treated with two types of calcium silicate materials and bioactive glass-based material extracts. Calcium silicate-based extracts had a significant increase in hDPSCs proliferation compared to bioactive glass-based extract. On day seven, there was a significant difference between the 3 groups in mineralized nodule formation. The biomineralization of bioactive glass-based material on pulp stem cells was evaluated and it was found that the bioactive glass-based material was similar to, yet more cytotoxic to hDPSCs than, light cure calcium silicate and calcium hydroxide^16^.

A systematic review^17^ aimed to collect different direct and indirect methods of using hydraulic calcium silicate cement in both in vitro and in vivo studies, and to determine if there was any superiority to indirect methods when inspected for biocompatibility, regeneration and differentiation abilities. The conclusion was that Biodentine and MTA were similar when they were evaluated for viability, proliferation, odontogenesis and osteogenesis of stem cells in vitro. In addition, they both had superior outcomes to various other commercially available hydraulic calcium silicate cements in equally direct and indirect methods. Permitting hydraulic calcium silicate cements to set for at least 24 h of incubation before their application results in the most anticipated outcomes.

With a wide variety of DPC biomaterials and the continuous development of new ones, little information is available about their biological behavior. Newly introduced experimental materials should undergo a series of in vitro tests to provide an initial idea about their performance. Thus, it is important to analyze the biological information related to dental pulp capping materials. This study aimed to evaluate the effect of four bioactive capping materials’ extracts on the proliferation and osteogenic differentiation of hDPSCs. The null hypotheses were that there is no significant difference in the effect of the four materials on both the proliferation and osteogenic differentiation of hDPSCs.

## Materials and methods

The four DPC materials evaluated in this study are presented in Table 1: calcium silicate material (Harvard MTA Universal- Harvard Dental International Gmbh, Hoppegartin, Germany), calcium zirconia complex (RetroMTA- BioMTA, Daejeon, Korea), bioactive glass-based material (ACTIVA Bioactive Base/Liner- PULPDENT Corporation, Watertown, USA) and MCP-based material (Experimental Pulpcap with 2% MCP-PULPDENT Corporation, Watertown, USA).

**Table 1:** Pulp capping materials used in this study.

Materials	Manufacturer	Product description	Manufacturers’ instructions	Lot
Harvard MTA Universal	Harvard dental International Gmbh, Hoppegartin, Germany	Powder and Liquid material contains Mixture of mineral oxides and bismuth oxide	Powder: Liquid mixing ratio 2.6:1 Mixing time: 30 s Working time: 3 min	91,805,537
RetroMTA	BioMTA, Daejeon, Korea	Powder and Liquid material contains Calcium carbonate, silicon dioxide, aluminum oxide, calcium zirconia complex	Premeasured powder/liquid Mixing time: 20 s Setting time: 2 min	RMBJ05D03
ACTIVA Bioactive Base/Liner	PULPDENT corporation, Watertown, USA	Dual cure material with a blend of diutherane and other methacrylates, polyacrylic acid, amorphous silica, sodium fluoride no Bisphenol A, No Bis-GMA and no BPA derivatives	Two-paste system in auto mix syringe Light cure setting time:20 s Initial self-cure setting time is 3 min	200,923
Experimental Pulpcap with 2% MCP	PULPDENT corporation, Watertown, USA	Light cure urethane-based resin with a modified calcium phosphate component No Bis-GMA, no Bisphenol A, no BPA derivatives, and no Fluoride	Ready-made paste applied directly Light cure for 10 s	200,910

Proliferation and osteogenic differentiation were performed using hDPSCs with the material extract of the four tested pulp capping materials at a ratio of 1:1 to culture media. The hDPSCs line was purchased from Global Research Center, Cairo, Egypt. The SciRAP guidelines for in vitro experiments were followed in conducting the current study (http://www.scirap.org).

### Preparation of extracts

Samples of the materials were prepared according to their manufacturers’ instructions in Teflon rings (5 mm in diameter, 2 mm high) to get a disc-shaped specimen under aseptic conditions in a class II biological safety cabinet flow hood. Each specimen was incubated in a humid incubator at 37 °C and 5% CO_2_ for 24 h to ensure thorough setting of the material. Both surfaces of each disc were subjected to ultraviolet light for 60 min using a 365 nm UV Lamp in order to sterile the specimens. Gilmore needle was used to confirm material setting before performing the test. Each material disc was placed into one insert of a 6-well plate. The discs were covered with Dulbecco’s Modified Eagle Medium (DMEM) (Gibco, Thermosientific, Germany) and incubated in a laminar hood at 37 °C in a dark atmosphere for 24 h. Following ISO 10993-12 guidelines, a ratio of contact area of the discs/medium of 1.25 cm^2^/ml was used. Subsequently, extracts of the test specimens were prepared at a 1:1 ratio^18^.

### Study design

In the MTT assay, the cells treated with complete media represent the negative control group. Cells cultured in osteogenic media (OM) represent the positive control group. Meanwhile, cells cultured on the tested extracts represent the tested groups. In alizarin red s (ARS) test, osteoblast differentiation media (ODM) served as the negative control and cells cultured on the tested extracts represent the tested groups.

### MTT cell proliferation assay

In 96-well plates, the extracts were inserted and 1 × 10^4^ hDPSCs were seeded on the extracts. Cells are maintained in DMEM (Gibco, Thermosientific, Germany) containing 10% FBS (Gibco, Thermosientific, Germany) and 1% antibiotic and an antimycotic solution having 10,000 IU/mL of penicillin G sodium, 10,000 µg/mL of streptomycin, and 25 µg/mL of amphotericin B (Gibco, Thermo Scientific, Germany). The cells treated with complete media represent the negative control group. Cells cultured in (OM) “alpha MEM supplemented with 100 nM of dexamethasone, 200 µM of ascorbic acid, and 10 mM of glycerol 2-phosphate” represent the positive control group. Meanwhile, cells cultured on the tested extracts represent the tested groups. The culture plate was incubated at 37 °C in an atmosphere of 5% CO_2_ for 4 days. Each condition was assessed in triplicate in three independent assays.

At the end of incubation time, the cell proliferation assay was performed using the Vybrant® MTT Cell Proliferation Assay Kit (cat no: M6494, Thermo Fisher, Germany). 100µL of media was removed and replaced by new media. Twenty µL of 4,5- dimethylthiazol-2-yl-2,5-diphenyltetrazolium bromide (MTT) solution (1 mg/mL) (Invitrogen, Thermo Scientific, Germany) was added to each well. The plates were incubated at 37 °C and 5% CO2 for 4 h. Finally, the MTT solution was removed and 100 µL of sodium dodecyl sulfate with hydrochloric acid (SDS-HCL) was added to the wells. Cell viability was determined by measuring the optical density (OD) at 570 nm on a spectrophotometer (ELx 800; Bio-Tek Instruments Inc., Winooski, VT, USA)^7,19^.

### Assessment of osteogenic differentiation via the Alizarin red assay

#### Alizarin red S staining

To evaluate calcium deposition in differentiated odontoblasts, 5 × 10^3^ cells were seeded in osteogenic media supplemented with 100 nM of dexamethasone, 200 µM of ascorbic acid, and 10 mM of glycerol 2-phosphate, along with 1% antibiotic and antimycotic solution having 10,000 IU/mL of penicillin G sodium, 10,000 µg/mL of streptomycin, and 25 µg/mL of amphotericin B (Gibco, Thermo scientific, Germany). Cells were maintained for one week at 37 °C and 5% CO_2_.

After incubation, the cultured medium was removed, and the cells were washed three times with 1X PBS. Subsequently, the cells were fixed with 4% formaldehyde (Merck Millipore, USA) for 15 min at room temperature, followed by three washes with deionized water. One mL of 40 mM (2%) Alizarin Red stain (ARS) at pH 4.2 was added and incubated for 30 min at room temperature with shaking^20^. The cells were then washed with deionized water five times and examined under a microscope using the LABOMED Trinocular inverted phase contrast microscope model TCM400, and the Atlas 16MP Cmos USB Camera with PixelPro 3.0 software (LABOMED, USA).

#### Photometric quantification of the Alizarin red S concentration

The photometric analyses were conducted as follows in 96-well plates: 50 µL of 10% acetic acid was added to the cells, which were then detached and transferred into a 1.5 mL Eppendorf tube. The tubes were incubated for 10 min at 85 °C and shaken at 750 rpm, followed by transfer to ice for 5 min. After centrifugation for 10 min at a maximum speed, at 4 °C, 35 µL of the supernatant was transferred to a new 1.5 mL Eppendorf tube. Subsequently, 13 µL of ammonium hydroxide (10% Alfa Aesar, Tewksbury, MA, USA) was added. Next, 40 µL of the suspension was transferred to a 96-well plate, and the absorbance of solubilized calcium-bound alizarin red S was measured at 405 nm using a TECAN microplate reader (TECAN, Männedorf, Switzerland).

**Fig. 1:**
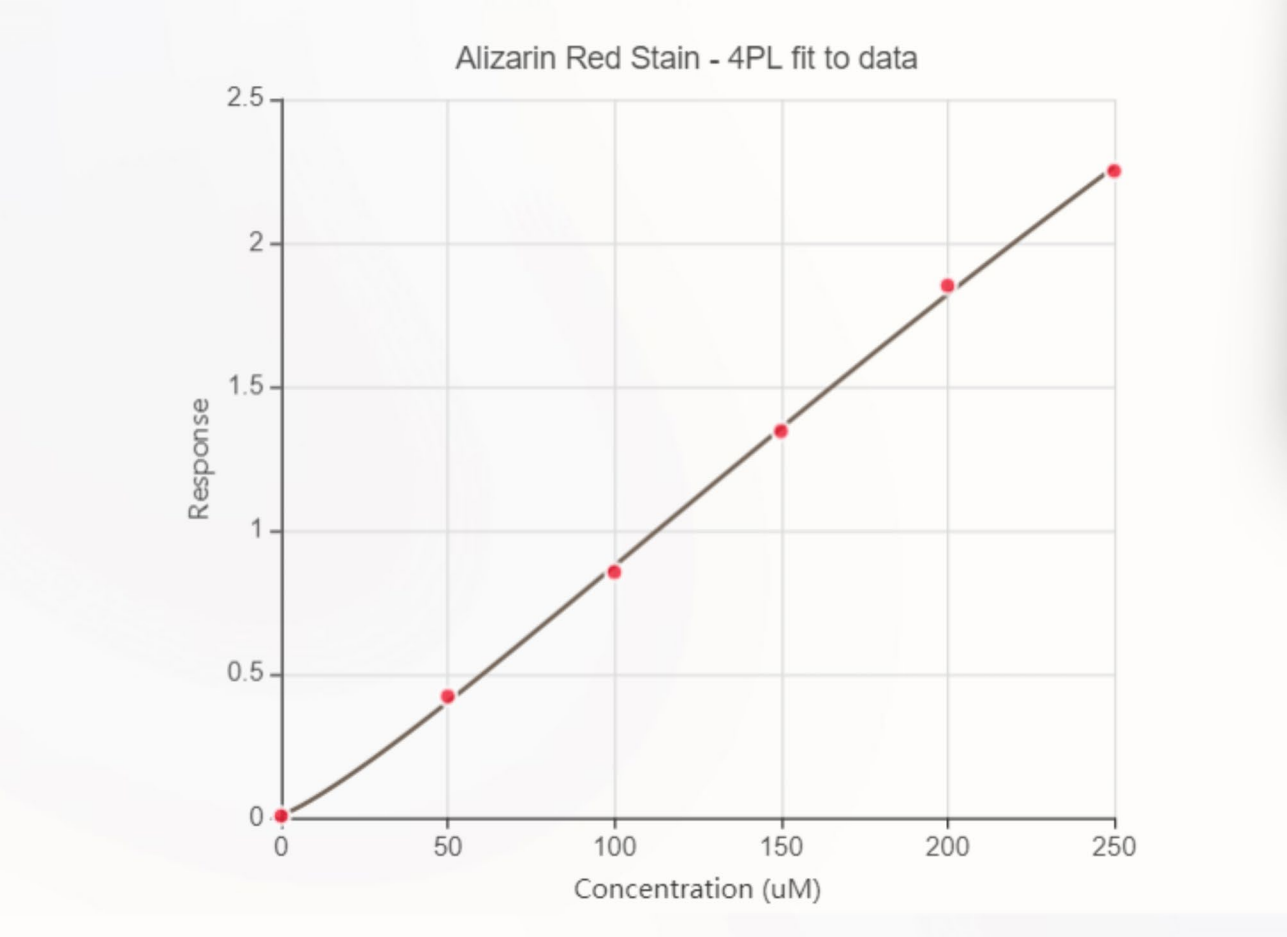
XY plot illustrating the standard curve for ARS, the data was plotted as the OD 450 of each standard solution (Y) vs. the respective concentration of the standard solution (X).

The concentrations of alizarin red S were determined mathematically using a standard curve (the data was plotted as the OD 450 of each standard solution (Y) vs. the respective concentration of the standard solution (X)). To prepare the standard curve, 100 µL of alizarin red S (40 mM) was mixed with 900 µL of a hydrochloric acid water solution (pH 4), and a dilution series was created in a 1:2 ratio to obtain different concentrations of alizarin red S (range: 31.3–200 µM), including a blank consisting of a hydrochloric acid water solution (pH 4) (Fig. 1).

The experimental procedure for quantifying alizarin red S concentrations in 24-well plates was identical, with the following volumes of substances used: 250 µL of 10% acetic acid added to the cells, 200 µL of the supernatant transferred to a new 1.5 mL Eppendorf tube, 75 µL of ammonium hydroxide (10%) added, and 50 µL of the suspension transferred to a 96-well plate for photometric measurement. The H-score was calculated as follows: (1 × percentage of weak staining) + (2 × percentage of moderate staining) + (3 × percentage of strong staining) within the target region, ranging from 0 to 300^21^.

The hDPSCs cells were cultured in four different osteogenic media to induce osteogenesis for seven days. Each condition was assessed in triplicate in three independent assays. ODM served as a negative control. Representative pictures of transmitted light microscopy at 20× magnification were captured. The scale bar was 50 μm (LABOMED Trinocular inverted phase contrast microscope model TCM400 with the Atlas 16MP Cmos USB Camera with PixelPro 3.0 software (LABOMED, USA)^22^.

### Statistical analysis

Each experimental condition was carried out three times and assessed in three independent experiments. Data was expressed as mean ± standard deviation (SD). The homogeneity of variance and normal distribution of the data were confirmed. SPSS software (IBM Corp, USA) was used to perform the parameter correlation. The data of proliferation and osteogenic differentiation were analyzed using one-way ANOVA followed by Tukey’s post hoc multiple comparison test. The *p* ≤ 0.05 was considered significant to analyze the differences amongst the means of groups.

## Results

### MTT cell proliferation assay results

**Fig. 2:**
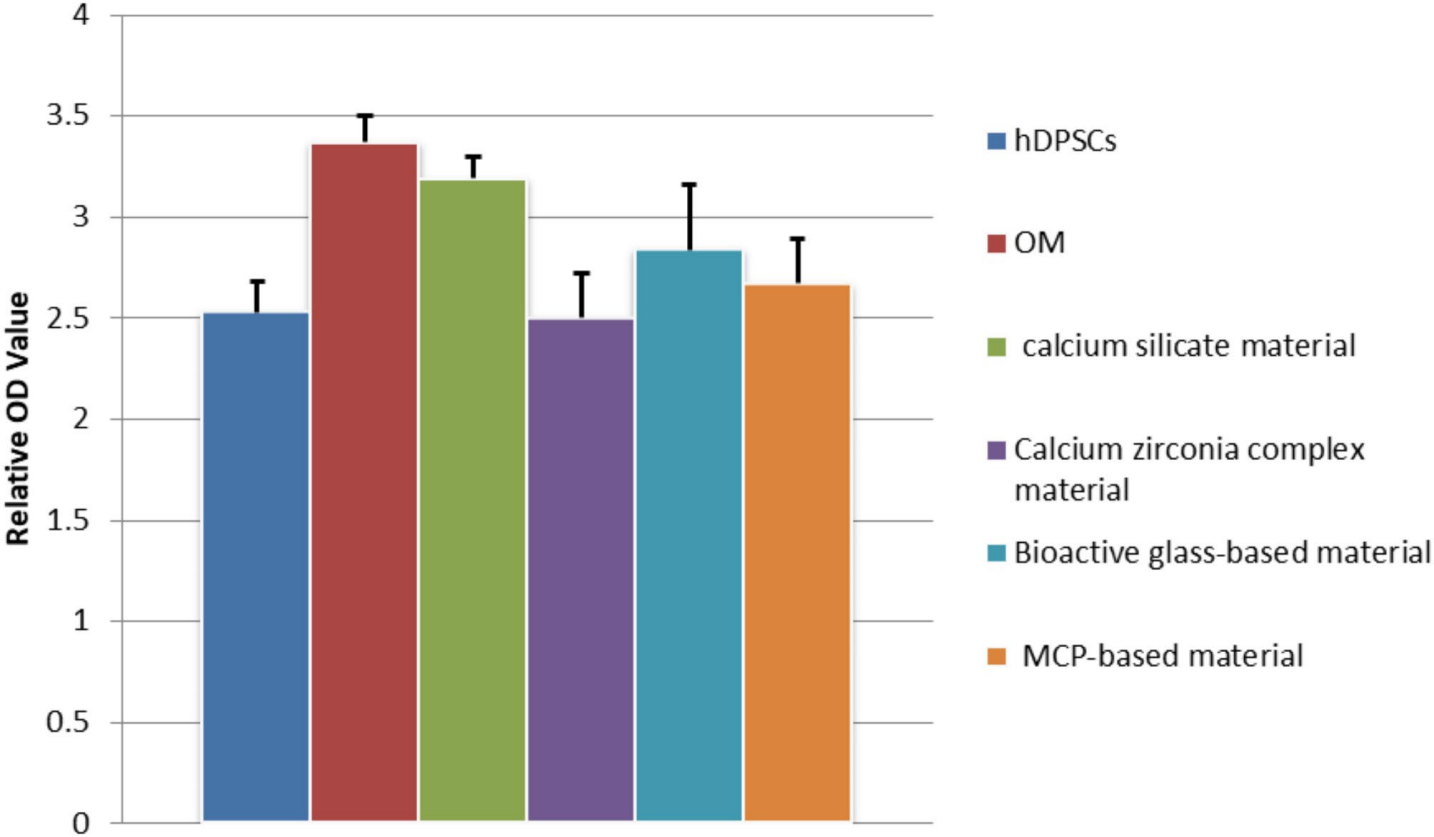
Relative OD for hDPSCs cells treated with four extracts.

The DPC materials significantly influenced the cellular proliferation of hDPSCs (*p* ≤ 0.05). Osteogenic media (OM) was significantly higher than hDPSCs, calcium zirconia complex and MCP-based material (*p* ≤ 0.05). Calcium silicate material showed significant increase in OD compared to hDPSCs and calcium zirconia complex (*p* ≤ 0.05). Calcium silicate material showed the highest value of OD among the four tested materials. Calcium zirconia complex showed the lowest value of OD among the four tested materials. There was no significant difference between calcium silicate material, bioactive glass-based material, and OM (*p* ≤ 0.05). Also, no significant difference was found between bioactive glass-based material and MCP-based material (*p* ≤ 0.05) (Table 2) (Fig. 2).

**Table 2:** MTT assay results for hDPSCs cells treated with four extracts.

Groups	*n*	OD
hDPSCs	3	2.53 ± 0.15
OM	3	3.37 ± 0.13^a, d,f^
Calcium silicate material	3	3.19 ± 0.11^a^
Calcium zirconia complex material	3	2.50 ± 0.22^c^
Bioactive glass-based material	3	2.84 ± 0.32
MCP based material	3	2.67 ± 0.22

Values are means ± standard deviation.

Groups identified by different superscripts were significantly different at *p* < 0.05.

Assay is the experimental unit of the current study, *n* = 3.

Significant difference compared to corresponding ^a^hDPSCs, ^b^OM, ^c^calcium silicate, ^d^ Calcium zirconia complex, ^e^ Bioactive glass-based, and ^f^ MCP based group.

### Alizarin red staining (ARS) for matrix calcium deposition analysis

**Fig. 3:**
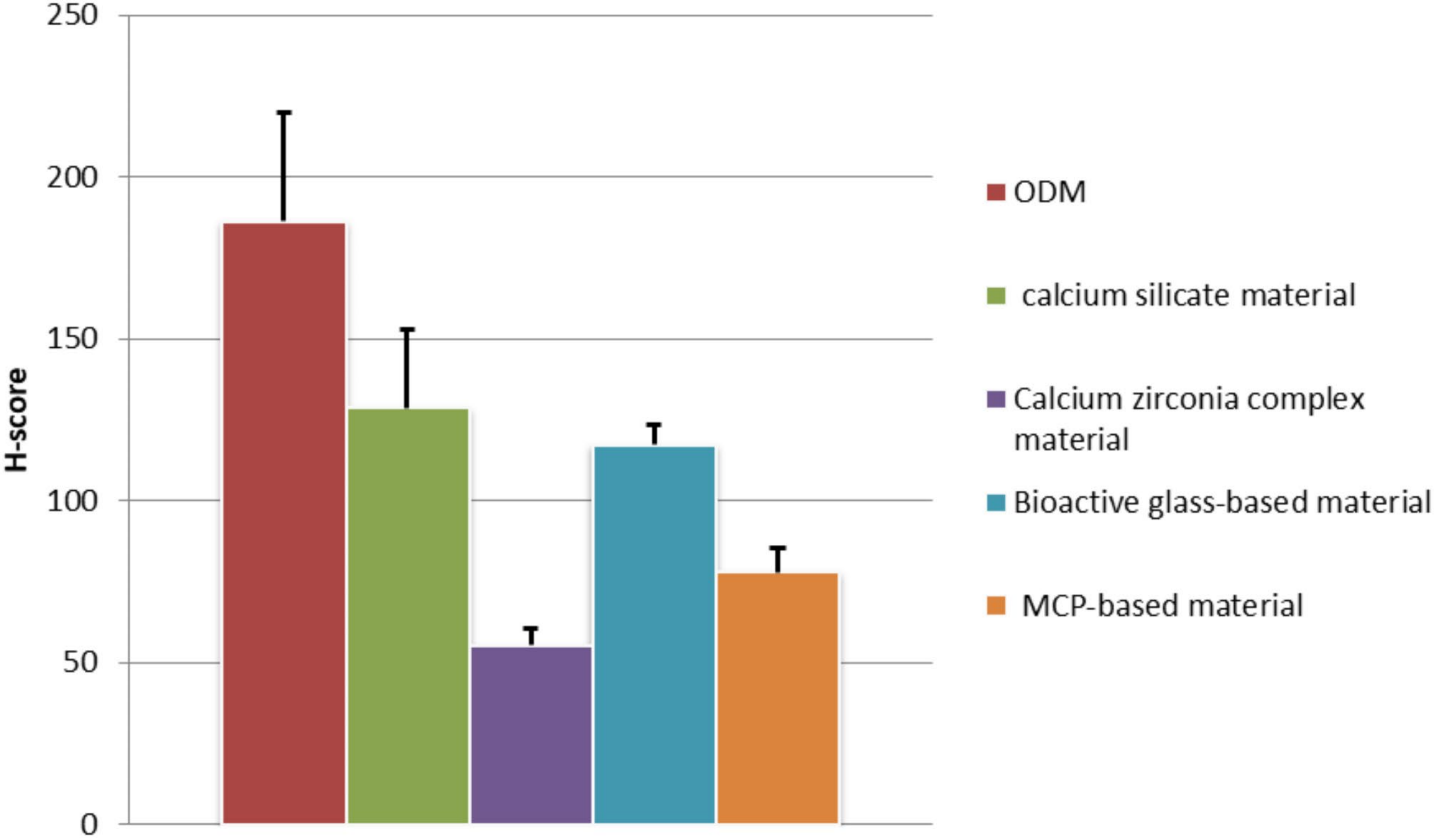
Reactive score for Calcium deposition in differentiated Odontoblast cells using Alizarin red stain (ARS).

The reactive score for Calcium deposition (H-score) in differentiated Odontoblast cells using (ARS) showed a significantly lower H-score of the four tested DPC materials than ODM (*p* ≤ 0.05). Calcium silicate material showed the highest H-score among the four tested materials. Calcium zirconia complex showed the lowest H-score among the four tested materials. The H-score of calcium zirconia complex was significantly lower than both calcium silicate material and bioactive glass-based material (*p* ≤ 0.05). There was no significant difference between calcium silicate material, bioactive glass-based material, and MCP-based material (*p* ≤ 0.05) (Table 3) (Fig. 3).

**Table 3:** Reactive score for calcium deposition in differentiated odontoblast cells using ARS.

Groups	*n*	H-Score
ODM	3	186.33 ± 33.62
Calcium silicate material	3	128.67 ± 24.19^a^
Calcium zirconia complex material	3	55.33 ± 5.03^ab^
Bioactive glass-based material	3	117.33 ± 6.11^ac^
MCP based material	3	78 ± 7.21^a^

Values are means ± standard deviation.

Groups identified by different superscripts were significantly different at *p* ≤ 0.05.

Assay is the experimental unit of the current study, *n* = 3.

Significant difference compared to corresponding ^a^ ODM, ^b^ calcium silicate, ^c^Calcium zirconia complex, ^**d**^ Bioactive glass-based and ^e^ MCP based group.

**Fig. 4:**
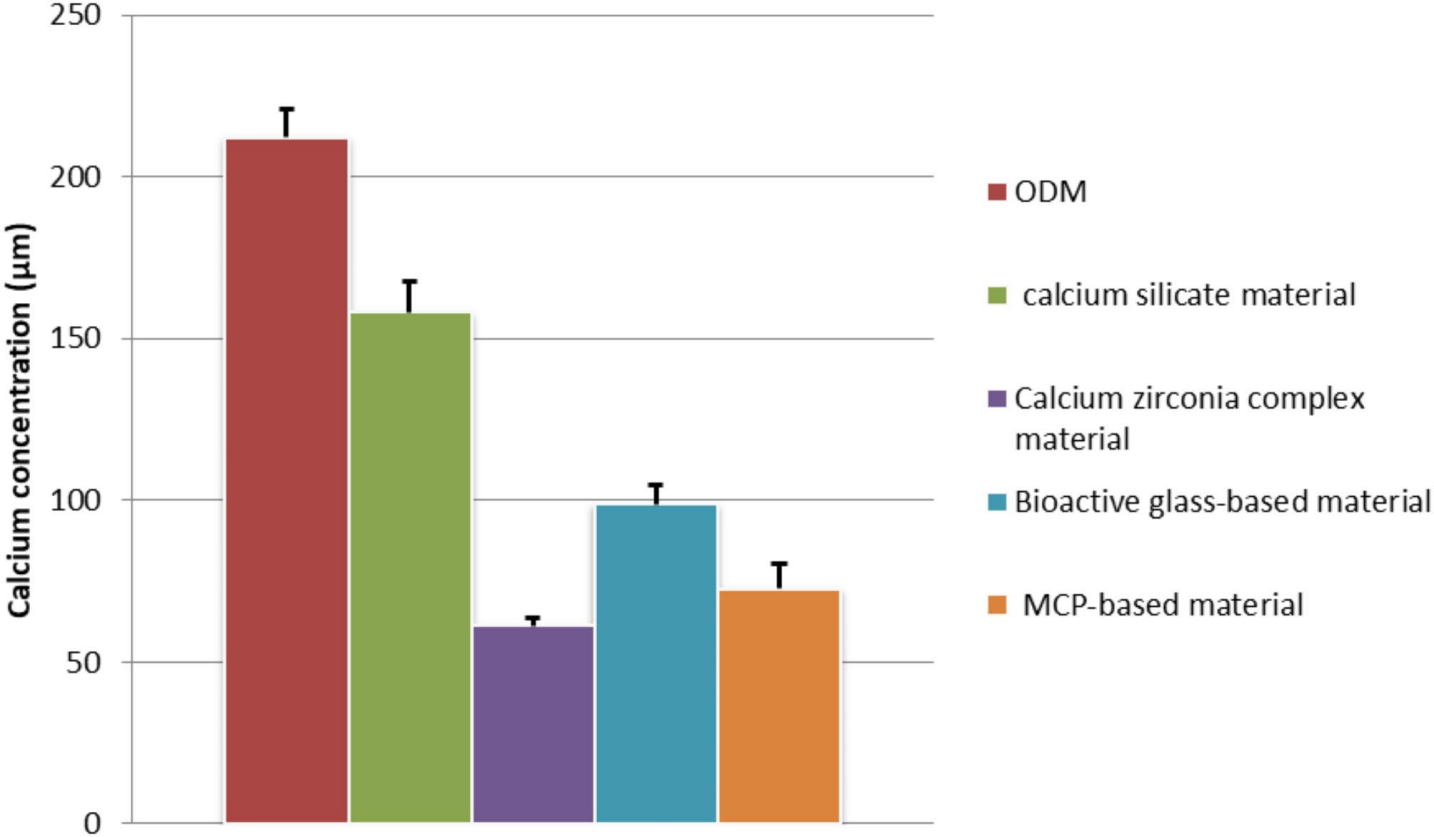
Calcium concentration in differentiated odontoblast cells using ARS.

**Fig. 5:**
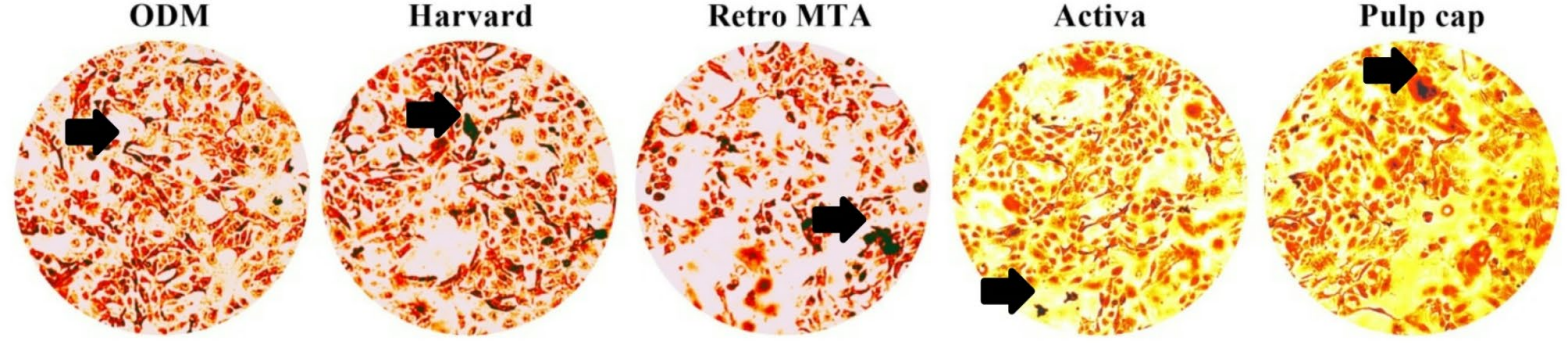
Mineralized deposits identified by ARS for hDPSCs grown in osteogenic medium after addition of the materials’ extract at day 7. Representative pictures of transmitted light microscopy at 20× magnification are shown. Scale bar is 50 μm.

Photometric Quantification of ARS Concentration in differentiated Odontoblast cells revealed significant difference between the calcium concentrations of the four tested materials and the ODM (*p* ≤ 0.05). Calcium silicate material showed the highest calcium concentration amongst the four tested material. Calcium zirconia complex showed the lowest calcium concentration among the four tested materials. The calcium concentration of calcium silicate material was significantly higher than the other three materials (*p* ≤ 0.05). Calcium zirconia complex showed significantly lower calcium concentration than both calcium silicate material and bioactive glass-based material (*p* ≤ 0.05) (Table 4) (Fig. 4). Mineralized deposits identified by ARS for hDPSCs grown in the osteogenic medium after the addition of the materials’ extract on day 7 were observed under a light microscope at 20× magnification in all groups (Fig. 5).

**Table 4:** Photometric quantification of ARS concentration in differentiated odontoblast cells.

Groups	No	OD at 405 nm	Calcium Concentration (µM)
ODM	3	1.93 ± 0.08	212.13 ± 8.69
calcium silicate material	3	1.43 ± 0.09^a^	158.12 ± 9.33^a^
Calcium zirconia complex material	3	0.51 ± 0.02^ab^	61.25 ± 2.33^ab^
Bioactive glass-based material	3	0.86 ± 0.06^abc^	98.82 ± 6.13^abc^
MCP based material	3	0.62 ± 0.07^ab^	72.68 ± 7.81^ab^

Values are means ± standard deviation.

Groups identified by different superscripts were significantly different at *p* ≤ 0.05.

Assay is the experimental unit of the current study, *n* = 3.

Significant difference compared to corresponding ^a^ ODM, ^b^ calcium silicate, ^c^ Calcium zirconia complex, ^d^ Bioactive glass-based and ^e^ MCP based group.

## Discussion

Biological evaluation of newly developed materials is paramount for decision-making during DPC procedures. The bioactive materials have a major influence on the outcome of DPC procedure. The aim of using a DPC material is to stimulate the undifferentiated dental pulp stem cells to differentiate into odontoblast-like cells and form reparative dentin to protect the pulp^23^.

Stem cell studies are reliable methods to evaluate the cytotoxicity, proliferation and osteogenic differentiation of newly developed materials. The MTT assay is one of the most common and reliable tests to evaluate cell viability and proliferation. Reduction of the MTT reagent into formazen occurs in metabolically active cells^24^. The MTT assay is fast, simple, truthful and cheap^25^. In order to further evaluate the efficacy of DPC material, osteogenic differentiation should be tested. The ARS test is a reliable method to assess extracellular calcium deposition^7^.

The materials used in the current study represent a variety of materials used in DPC. Calcium silicate cements based on the chemical composition of conventional MTA were introduced with the advantages of MTA and without its drawbacks; their setting time is mostly shorter and the color changes seem to be less noticeable^7,26,27^. The powder components of calcium zirconia complex material are calcium carbonate, silicon dioxide, aluminum oxide and calcium zirconium complex^8^. The material is prepared by mixing the powder with water manually, which leads to calcium hydroxide formation and induction of hard tissue formation. The initial pH of hydraulic calcium zirconia complex material is 9.9 then it decreases to 7.9 after one week. These levels are comparable with MTA^9^. Both bioactive glass-based material and MCP-based material setting reactions were initiated with light curing devices as they contain urethane-based resin. This allows the immediate application of next step.

In the current study, MTT assay results showed that calcium silicate material had the highest OD with no significant difference with the positive control group (OM) (*p* ≤ 0.05). This may be caused by the mineral oxides component that may attract stem cells and stimulate their differentiation. This process could be attributed to high amount of released calcium and silicon ions, in addition to the absence of aluminum which stimulated mitogen-activated protein kinase (MAPK) cascade and autophagy encouraged the osteo/odontogenic differentiation of hDPSCs^7^. This was in agreement with Michel et al.^28^ who found that calcium silicate material allowed the proliferation of gingival fibroblasts and alveolar osteoblasts. However, Youssef and Elsherief^29^ found that calcium silicate material induced the proliferation of human dental fibroblasts after one day while it was cytotoxic after 4 days. Lozano-Guillen et al.^30^ Study found that the undiluted (1:1) extracts of different calcium silicate materials demonstrated decreased cell viability. The study suggested that excessive presence of calcium ions in the intracellular spaces associated with the high alkalinity may lead to mitochondrial dysfunction which affects the results of the MTT assay.

Calcium zirconia complex had the lowest OD among all groups and was significantly lower than both OM and calcium silicate material (*p* ≤ 0.05). The lower result of calcium zirconia complex material could be attributed to differences in the manufacturing process and its components, that mainly associated wth short setting time and absence of heavy metals^8^. However, Chung et al.^31^ found that calcium zirconia complex material didn’t decrease cell viability of lipopolysaccharide-stimulated hDPSCs. On the contrary, Souza et al.^9^ found that calcium zirconia complex material had significantly higher cell viability than the positive control. The conflicting results may be caused by the different viability assays used. Souza et al. used a multiparametric assay rather than MTT assay. MTT assay measurements depend on mitochondrial metabolic activity rate. However, excess mitochondrial activity may be a sign of increased cellular stress^32^. Another study aimed to evaluate the biocompatibility three calcium silicate- zirconia-based sealers on human periodontal ligaments stem cells found that bioceramic sealers showed enhanced cell viability over time^33^.

Both bioactive glass-based material and MCP-based material extracts had significantly lower OD than the OM (*p* ≤ 0.05). The cytotoxic effect of bioactive glass-based material may be attributed to its fluoride release property^6,34,35^. Kanjevac et al.^36^ suggested a direct correlation between material cytotoxicity and the fluoride release of some restorative materials. Jun et al.^16^ found that bioactive glass-based material was more cytotoxic compared to MTA-like and CH capping materials. Also, Abou ElReash et al.^7^ study showed that MTA and bioceramic-based root canal sealer had a significant increase in hDPSCs proliferation compared to bioactive glass-based material. On the other hand, Lopez-Garcia et al.^37^ noticed that the metabolic activity of cells subjected to bioactive glass-based material increased at 24, 48 and 72-h, indicating lower cytotoxicity.

The lower OD of MCP-based material may be attributed to the concentration of the MCP component (2%). A study examined the effect of nanohydroxy appetite (naHAp) concentration on the proliferation of dental pulp stem cells (DPSCs). It showed that 10% and 30% concentrations had no significant effect on proliferation of DPSCs^38^.

Analysis of matrix calcium deposition revealed that all materials stimulated osteogenic differentiation and calcium deposition. Calcium concentration of calcium silicate material was significantly higher than the other three materials (*p* ≤ 0.05). Formation of calcified nodules could be linked to cytotoxicity of material elutes^6^. This could be attributed to the setting mechanism of calcium silicate cements starts after hydration by water during the mixing process. The formed calcium silicate hydrate by-products formed after mixing have many phases. These stages including porous colloidal CSH gel and radial acicular CSH crystals, rhombohedral crystals of portlandite, needlelike crystals of ettringite which is a hexacalcium alumina-tetrisulphate hydrate, and calcium mono- sulfoaluminate or calcium mono-carboaluminate. Moisture plays a major role in initiation of apatite formation (bioactivity) and nucleation process of calcium ions on the surface of hydrated calcium silicate particles, it goes through defined stages starting from rapid dissolution of calcium ions after the solid–liquid interface formed on the cement particles forming portlandite.

Furthermore, inhibition of proliferation may upturn the likelihood of osteogenic differentiation and calcified nodules deposition^39^.

Subjecting the pulp to MTA and calcium zirconia complex material caused release of differentiation markers and produced the fencing pattern of odontoblast cells^8^. It was suggested that the mineralization potential of calcium silicate materials is due to their ability to release calcium and hydroxide ions thus stimulating the formation of calcium phosphate deposits^40^.

In accordance with the current study, Abou ElReash et al. study^7^ showed that bioactive glass-based material extract had less influence on hDPSCs mineralized nodules formation. This can be attributed to the response of bioactive glass-based material to pH cycles and fluoride release and recharge^41^. Yoshida et al.^38^ suggested that naHAp helped the formation of mineralized deposits by DPSCs, which was dependent on the ratio of naHAp based on the result of ARS staining.

Based on the results of the current study, the null hypotheses were rejected. The current study assessed the viability and osteogenic differentiation of hDPSCs by four bioactive materials, which are essential for DPC. The present study had few limitations; mainly it is an in vitro study. It is recommended to conduct in vivo study to assess the regenerative properties of the materials and their abilities on dynamic tissues. Also, further laboratory tests should be performed regarding the experimental material - antimicrobial activity and physical properties- to allow a better understanding of its behavior.

## Conclusions

Calcium silicate-based materials exhibited the most reliable performance regarding the proliferation and osteogenic differentiation of hDPSCs. Newly introduced resin-based materials showed acceptable results but need further investigation. The results act as supporting preliminary evidence for the use of tested materials in DPC until additional clinical supporting evidence is reported.

## Data Availability

The datasets generated and/or analyzed during the current study are not publicly available but are available from the corresponding author upon reasonable request.
